# High-Fat Diet Leads to Reduced Protein O-GlcNAcylation and Mitochondrial Defects Promoting the Development of Alzheimer’s Disease Signatures

**DOI:** 10.3390/ijms22073746

**Published:** 2021-04-03

**Authors:** Ilaria Zuliani, Chiara Lanzillotta, Antonella Tramutola, Eugenio Barone, Marzia Perluigi, Serena Rinaldo, Alessio Paone, Francesca Cutruzzolà, Francesco Bellanti, Matteo Spinelli, Francesca Natale, Salvatore Fusco, Claudio Grassi, Fabio Di Domenico

**Affiliations:** 1 Laboratory affiliated to Istituto Pasteur Italia-Fondazione Cenci Bolognetti, Department of Biochemical Sciences “A. Rossi Fanelli”, Sapienza University of Rome, 00185 Rome, Italy; ilaria.zuliani@uniroma1.it (I.Z.); chiara.lanzillotta@uniroma1.it (C.L.); antonella.tramutola@uniroma1.it (A.T.); eugenio.barone@uniroma1.it (E.B.); marzia.perluigi@uniroma1.it (M.P.); serena.rinaldo@uniroma1.it (S.R.); alessio.paone@uniroma1.it (A.P.); francesca.cutruzzola@uniroma1.it (F.C.); 2 Department of Medical and Surgical Sciences, University of Foggia, 71122 Foggia, Italy; francesco.bellanti@unifg.it; 3 Department of Neuroscience, Università Cattolica del Sacro Cuore, 00168 Rome, Italy; matteo.spinelli@unicatt.it (M.S.); francesca.natale1@unicatt.it (F.N.); salvatore.fusco@unicatt.it (S.F.); claudio.grassi@unicatt.it (C.G.); 4 Fondazione Policlinico Universitario Agostino Gemelli IRCCS, 00168 Rome, Italy

**Keywords:** high-fat diet, brain insulin resistance, neurodegeneration, protein O-GlcNAcylation, mitochondria

## Abstract

The disturbance of protein O-GlcNAcylation is emerging as a possible link between altered brain metabolism and the progression of neurodegeneration. As observed in brains with Alzheimer’s disease (AD), flaws of the cerebral glucose uptake translate into reduced protein O-GlcNAcylation, which promote the formation of pathological hallmarks. A high-fat diet (HFD) is known to foster metabolic dysregulation and insulin resistance in the brain and such effects have been associated with the reduction of cognitive performances. Remarkably, a significant role in HFD-related cognitive decline might be played by aberrant protein O-GlcNAcylation by triggering the development of AD signature and mitochondrial impairment. Our data support the impairment of total protein O-GlcNAcylation profile both in the brain of mice subjected to a 6-week high-fat-diet (HFD) and in our in vitro transposition on SH-SY5Y cells. The reduction of protein O-GlcNAcylation was associated with the development of insulin resistance, induced by overfeeding (i.e., defective insulin signaling and reduced mitochondrial activity), which promoted the dysregulation of the hexosamine biosynthetic pathway (HBP) flux, through the AMPK-driven reduction of GFAT1 activation. Further, we observed that a HFD induced the selective impairment of O-GlcNAcylated-tau and of O-GlcNAcylated-Complex I subunit NDUFB8, thus resulting in tau toxicity and reduced respiratory chain functionality respectively, highlighting the involvement of this posttranslational modification in the neurodegenerative process.

## 1. Introduction

Metabolic syndrome is a series of complex metabolic disorders centering on insulin resistance, obesity, hyperglycemia, hypertension, and dyslipidemia, which represents a high-risk factor for a number of pathological conditions including Type-2 diabetes mellitus (T2DM), nonalcoholic steatohepatitis, coronary heart disease, stroke, and cognitive decline [1]. T2DM accounts for 90% of all reported diabetes cases [2,3] and it is attributed to the interaction of multiple genetic and environmental factors. One of the environmental risks is the modern lifestyle with the so-called Western diet, rich in refined carbohydrates, animal fats and edible oils, which leads to nutrient excess and promotes the development of insulin resistance [4]. Systemic and brain insulin resistance, as common features of Alzheimer’s disease (AD) and T2DM, play prominent roles in the development of cognitive dysfunction and dementia [5,6,7,8]. Epidemiologic studies indicate that long-term hyperinsulinemia and obesity, caused by dietary fat intake, are risk factors for dementia. Moreover, insulin administered to AD patients, by regulating glucose transport, energy metabolism, neuronal growth, and synaptic plasticity, improves memory formation [9,10,11]. Studies on mice fed with a high-fat diet (HFD), which mimicked a hypercaloric Western-style diet [12], thus leading to obesity and T2DM, demonstrated impaired performance in learning and memory tasks, alterations of synaptic integrity, altered CA1-related long-term depression (LTD) and long-term potentiation (LTP), and the development of AD pathological hallmarks [13,14,15,16]. However, the identification of the exact molecular mechanisms associating perturbations in the peripheral environment with brain function is unclear and effective treatments are lacking. Thus, there is an urgent need to further explore the molecular role of insulin resistance in order to find innovative strategies and key targets for the prevention and treatment of metabolic syndrome. In this scenario, as the nutrient-sensing mechanisms are essential for regulation of metabolism, it might be of great significance to explore the biological mechanisms that link insulin resistance, metabolic syndrome and neurodegeneration from the perspective of nutrient recognition and regulation.

In this regard, protein O-GlcNAcylation is widely recognized as an important cellular nutrient sensor and it serves as a key linkage between nutrient sensing, energy metabolism and signal transduction [17]. O-GlcNAcylation is a post-translational modification that dynamically modifies serine (Ser) and threonine (Thr) through their hydroxyl moieties on nuclear and cytoplasmic proteins [18]. O-GlcNAcylation is catalyzed by two key enzymes, namely, O-GlcNAc transferase (OGT) and O-GlcNAcase (OGA), which add/remove UDP-GlcNAc to/from Ser and Thr residues, respectively [19]. O-GlcNAc modification is dependent on the intracellular concentration of UDP-GlcNAc from the hexosamine biosynthetic pathway (HBP). The HBP integrates the metabolic information of nutrients, including carbohydrates, amino acids, fatty acids, and nucleic acids, in the process of UPD-GlcNAc synthesis [4]. Further, HBP regulates energy homeostasis by controlling the production of both insulin and leptin, which are hormones playing a critical role in regulating energy metabolism [20]. O-GlcNAcylation is tightly coupled to insulin resistance because hyperglycemia or hyperlipidemia-induced insulin resistance is closely related to altered HBP flux, and in turn, the subsequent aberrant O-GlcNAcylation modifies insulin signaling, glucose uptake, gluconeogenesis, glycogen, and fatty acid synthesis [21]. In this scenario, O-GlcNAcylation represents a key mechanism linking nutrient overload and insulin resistance, and their dysregulation might promote the transition from metabolic defects to chronic diseases such as T2DM and AD [22,23,24,25,26]. Indeed, obesity and peripheral hyperglycemia, by promoting insulin resistance and hypoglycemia at brain level, lead to decreased O-GlcNAcylation of APP and Tau and to increased production of toxic Aβ amyloid and Tau aggregates, which are hallmarks of AD [27,28,29,30,31]. Furthermore, the well-known involvement of O-GlcNAcylation in controlling protein localization, function, and interaction has acquired special interest with regards to APP. Indeed, this posttranslational modification (PTM) has been demonstrated to control APP maturation and trafficking, thus affecting the generation Aβ [32,33,34]. Moreover, mitochondrial dysfunction and the associated oxidative stress have been recognized as important biological mechanisms contributing to insulin resistance and diabetic complications [35,36,37,38]. As well, a number of studies demonstrated that impaired mitochondrial function plays a significant role in neurodegenerative diseases, supporting the notion that AD is primarily a metabolic disorder [14,39,40,41,42,43]. O-GlcNAcylation is known to regulate energy metabolism and the production of metabolic intermediates through the dynamic modulation of mitochondrial function, motility, and distribution. Past and present proteomics analysis revealed the presence of O-GlcNAc-modified mitochondrial proteins in different rodents’ organs (e.g., brain) supporting that the overexpression of OGT or OGA proteins alters mitochondrial proteome and severely affects mitochondrial morphology and metabolic processes [44,45,46]. Further, hyperglycemia was associated with the aberrant O-GlcNAcylation of mitochondrial protein and with the modulation of the electron transport chain activity, oxygen consumption rate, ATP production, and calcium uptake in cardiac myocytes [44,47,48]. Thus, aberrant protein O-GlcNAcylation and its regulation of mitochondrial proteins might represent the missing link between metabolic defects and neurodegeneration occurring in T2DM and AD. Therefore, the present study aimed to decipher the role of O-GlcNAcylation and associated mitochondrial abnormalities in mediating the development of AD signatures in HFD mice by exploring how nutrient excess leads to the alteration of both OGT/OGA cycle and of energy consumption/production, thus promoting the development of AD hallmarks.

## 2. Results

### 2.1. HFD Mice Show an Aberrant and Tissue-Specific O-GlcNAcylation Profile

In the present study, we aimed to explore the impact of nutrient overload in promoting putative changes of protein O-GlcNAcylation in the brain of mice fed with a diet rich in fat content (HFD) in comparison to aged-matched animals that received a standard diet (SD).

**Figure 1:**
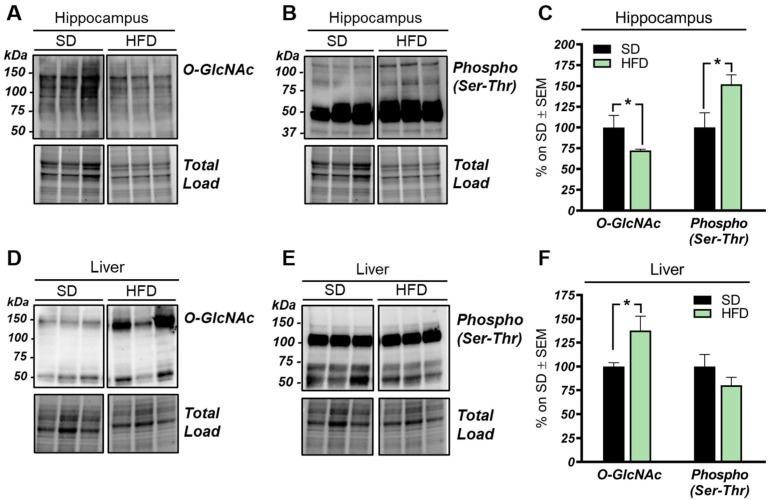
O-GlcNAcylation and phosphorylation profile in the hippocampus and liver from high-fat diet (HFD) mice. (**A–C**): O-GlcNAcylation/phosphorylation profile in the hippocampus from HFD mice compared to a respective standard diet (SD) animals. The reduction of protein O-GlcNAcylation in the hippocampus of HFD mice was in line with a mutual inverse increase in the global phosphorylation of serine and threonine residues compared to SD controls. Representative blots are reported in (**A**,**B**). (**D,E**): O-GlcNAcylation/phosphorylation profile in the liver of HFD mice compared to respective control group. Increased levels of O-GlcNAcylated proteins were observed in the liver of HFD compared to animals fed with a SD, confirming a global imbalance of O-GlcNAcylation homeostasis in peripheral organs of our model. A trend of increase was observed in the phosphorylation of serine and threonine of hepatic proteins from HFD in comparison to SD mice. Representative blots are reported in (**D**,**E**). Number of animals for each condition were as follow: *n* = 6/group for Western blot. All bar charts reported in (**C**,**F**) show mean ± SEM. * *p* < 0.05, using Student’s t test.

Our analysis of total O-GlcNAcylated protein levels in the hippocampus of HFD mice revealed a significant reduction if compared to the total O-GlcNAcylation levels observed in the same brain region from SD mice (Figure 1A–C; * *p* < 0.05, SD vs. HFD: −28%). In line with the well-known competition occurring between protein O-GlcNAcylation and phosphorylation [49,50,51], we also observed an increase of total protein serine/threonine phosphorylation (Figure 1A–C; * *p* < 0.02, SD vs. HFD: +52%). Since HFD is a well-established model of diet-induced obesity and insulin resistance [52], we also evaluated the O-GlcNAcylation of liver proteome from HFD mice. Hyperglycemia and dysfunctional insulin signaling have been associated, in peripheral organs, with increased levels of O-GlcNAcylated proteins [53,54]. In agreement, we observed a significant increase in the total O-GlcNAcylated protein levels in the liver of HFD mice in comparison to the control group (Figure 1D,F; * *p* < 0.02, SD vs. HFD: +38%). Meanwhile, in regards to protein phosphorylation, we demonstrated a slight nonsignificant decrease in serine/threonine in the liver from HFD mice compared to SD mice (Figure 1D–F; SD vs. HFD: −20%).

**Figure 2:**
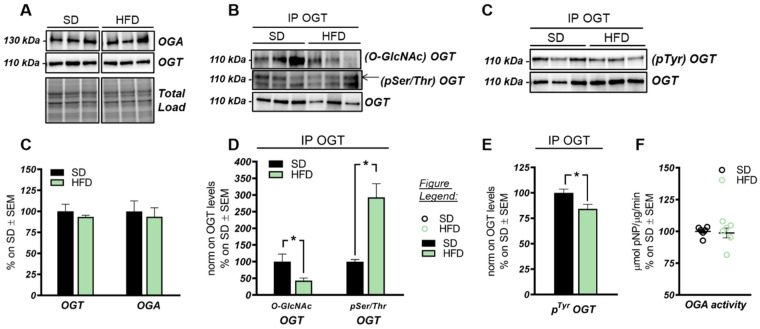
OGT/OGA functionality in the hippocampus of HFD mice. (**A**,**C**) OGT and OGA protein levels in HFD mice hippocampi. No relevant changes were observed in the protein expression levels of the enzymes controlling O-GlcNAc cycling. Representative blots are reported in (**A**). (**B**,**D**) Evaluation of OGT’s posttranslational modifications(PTMs) by immunoprecipitation analysis. A significant reduction in ^O-GlcNAc^OGT/OGT levels, together with a consistent increase in its ^pSer/Thr^OGT/OGT levels, were observed in the hippocampi of HFD mice in comparison to the same brain region from SD mice. Representative blots are reported in (**B**). (**C**,**E**) Evaluation of OGT phosphorylation on Tyr residues. A significant reduction was observed in HFD mice compared to respective SD group, suggesting an alteration of OGT functionality. Representative blots are reported in (**C**). (**F**): OGA activity assay. No changes were observed in OGA enzymatic activity between the two experimental groups. Number of animals for each condition were as follows: *n* = 6/group for Western blot and OGA assay analysis while *n* = 3/group was used for immunoprecipitation analysis. All bar charts reported in (**C**–**E**), and (**F**) show mean ± SEM. * *p* < 0.05 using Student’s t test.

**Figure 3:**
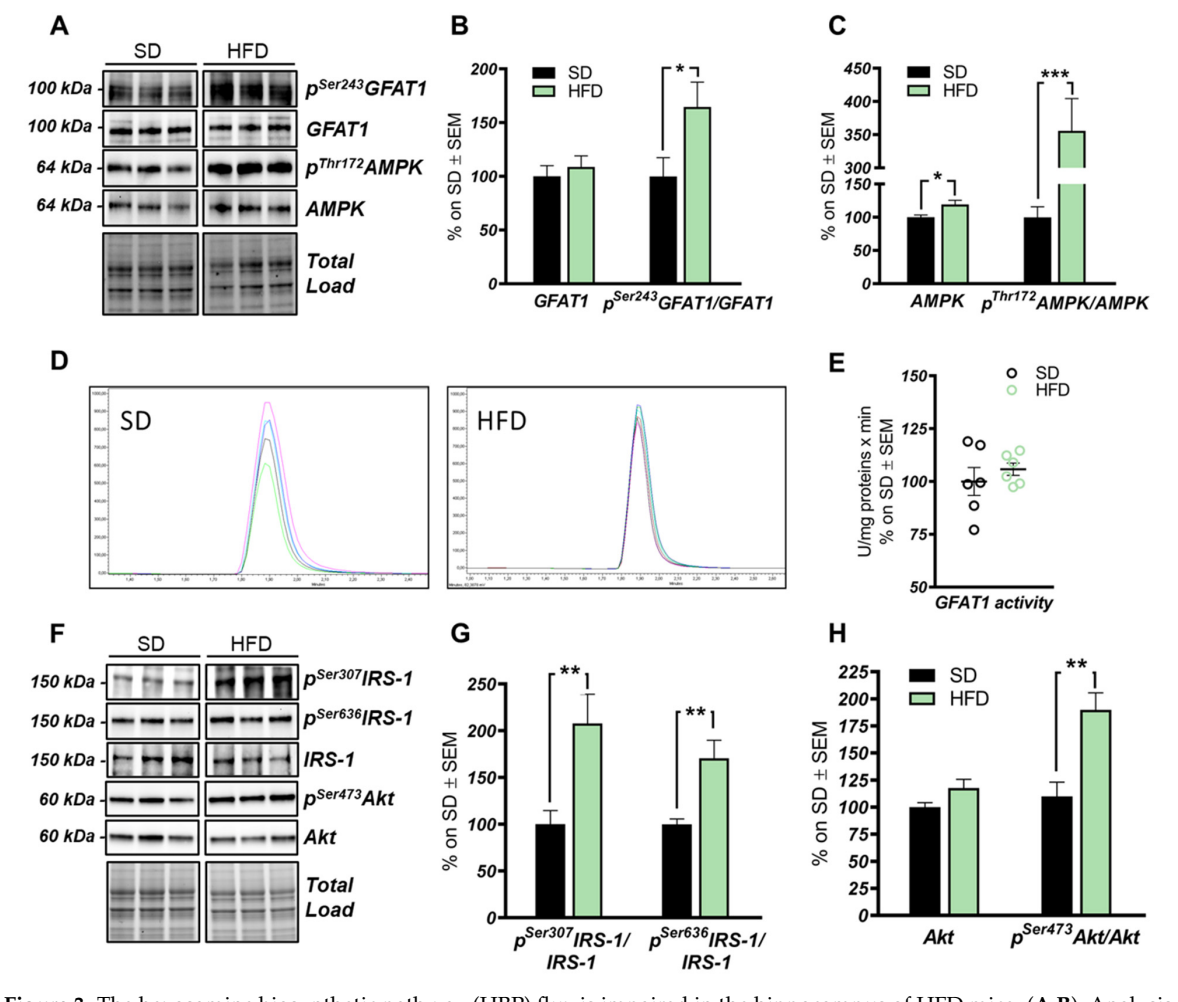
The hexosamine biosynthetic pathway (HBP) flux is impaired in the hippocampus of HFD mice. (**A**,**B**): Analysis of GFAT1 activation status in HFD mice compared to respective controls. A significantly increased p^Ser243^GFAT1/GFAT1 ratio was observed in HFD mice compared to SD, thus resulting in the inhibition of GFAT1 activity. Representative blots are reported in (**A**). (**A**,**C**) Analysis of AMPK activation status in HFD mice compared to respective SD animals. A significant increase in the AMPK protein levels was observed in HFD, together with a relevant increase in p^Thr172^AMPK/AMPK levels, thus resulting in increased AMPK activation. Representative blots are reported in (**A**). (**D**,**E**) GFAT1 activity assay. No relevant changes were observed in GFAT1’s ability to synthetize glucosamine-6-phosphate in vitro between the HFD group and SD mice. Representative spectra of GFAT1-synthetized glucosamine-6-phosphate for both HFD and SD animals are reported in (**E**). (**F**,**G**) Analysis of the IRS-1 activation state in the hippocampi of HFD mice compared to SD-fed animals. A relevant increase in both p^Ser307^IRS-1/IRS-1 and p^Ser636^IRS-1/IRS-1 levels was observed, suggesting a reduction in IRS-1 activation in HFD mice compared to SD. Representative blots are reported in (**F**). (**F**,**H**) Analysis of Akt activation status in HFD mice compared to respective controls. p^Ser473^Akt/Akt ratio was significantly increased in the HFD hippocampal region compared to the same brain area of SD mice. Number of animals for each condition were as follow: *n* = 6/group for both Western blot analysis and GFAT1 activity assay. All bar charts reported in (**B**,**C**) and (**G**,**H**) show mean ± SEM. * *p* < 0.05, ** *p* < 0.01, *** *p* < 0.001 using Student’s t test.

These data suggest that the O-GlcNAcylation profile was completely opposite to that examined in the hippocampus and that a direct competition between O-GlcNAcylation/phosphorylation may only occur at brain level, while peripheral alterations are mainly associated with the increase of protein O-GlcNAcylation. These results follow previous data collected in Down syndrome mice [55] and are in line with the notion that the aberrant increase of peripheral O-GlcNAcylation levels might be related to insulin resistance and to hyperglycemia-induced glucose toxicity [56]. Afterwards, we evaluated potential alterations occurring on the O-GlcNAc enzymatic machinery with the aim of assessing whether the reduced levels of O-GlcNAcylated proteins in the hippocampus of HFD mice could be the result of the altered OGT/OGA functionality. In details, we did not observe any relevant changes, either in OGT or OGA protein expression levels, in the hippocampus of our HFD model (Figure 2A,C). Considering the role of OGT as a sensor of cellular metabolic state [57,58], we further analyzed OGT PTMs through immunoprecipitation to evaluate the effects of nutrient surplus on its functionality. Interestingly, we observed a significant reduction of ^O-GlcNAc^OGT levels in the hippocampi of mice with a HFD (Figure 2B,D; * *p* < 0.05, SD vs. HFD: −57%), together with a substantial increase of ^pSer/Thr^OGT levels in our HFD model compared to the same brain region of SD animals (Figure 2B,D; * *p* < 0.05, SD vs. HFD: +193%). The aberrant changes of OGT Ser/Thr PTMs observed by us may suggest a potential imbalance of the O-GlcNAcylation/phosphorylation ratio, which may involve specific catalytic and/or regulatory residues of OGT. Despite the fact that involvement of serine residues was reported to regulate OGT function, studies by Hart laboratory supported the idea that OGT activity is mostly regulated by the phosphorylation of tyrosine residues [59]. Our analysis of total ^pTyr^OGT levels demonstrated a significant reduction in HFD mice hippocampi (Figure 3C,E; * *p* < 0.05, SD vs. HFD: −15%), supporting the notion of altered OGT’s enzymatic ability to transfer O-GlcNAc moiety. In contrast, OGA did not show any variation in its enzymatic functionality (Figure 2F), excluding a role for this enzyme in the reduction of O-GlcNAcylation levels in HFD mice.

### 2.2. The HBP Flux Is Impaired in HFD Mice Compared to SD

The HBP pathway integrates multiple metabolic pathways into the synthesis of UDP-GlcNAc, providing feedback on overall cellular energy levels and nutrient availability [60]. In this scenario, increased flux of metabolites into the HBP have already been highlighted as a key point driving metabolic alterations in the skeletal muscle of a model of fat-induced insulin resistance [61]. GFAT1 is the rate-limiting enzyme that coordinates nutrients’ entrance into the HBP flux, and it is negatively regulated by AMPK upon its activation [62,63]. The analysis of the HFD hippocampus has demonstrated a significant increase of Ser243 inhibitory phosphorylation of GFAT1 compared to a respective control group (Figure 3A,B; * *p* < 0.05, SD vs. HFD: +65%). No relevant changes were observed in GFAT1 protein expression levels between the two groups (Figure 3A,B). In line with these data, HFD mice showed the increase of AMPK-activating phosphorylation on Thr172 compared to animals that have been fed with a SD (Figure 3A,C; *** *p* < 0.001, SD vs. HFD: +255%), together with a significant increase in AMPK protein levels in HFD mice (Figure 3A,C; * *p* < 0.05, SD vs. HFD: +20%). AMPK is known to inhibit GFAT1 activity under nutrient depletion or stress conditions via its phosphorylation on Ser243, in order to reduce the amount of nutrients entering the HBP flux [62,63,64]. Interestingly, by testing GFAT1’s ability to synthetize glucosamine-6-phosphate in vitro by HPLC, we did not observe significant changes between HFD hippocampal extract and respective SD controls (Figure 3D,E). Since the GFAT1 activity assay was carried out in a large excess of substrates [65], the reduced GFAT1 activation (measured as increased ^pSer243^GFAT1/GFAT1 levels) may result from reduced substrate availability in the intracellular environment. These findings suggest that HFD may lead to the reduction of nutrient uptake (i.e., glucose) in the brain, promoting AMPK-mediated inhibition of GFAT1, finally resulting in reduced synthesis of UDP-GlcNAc through the HBP and decreased protein O-GlcNAcylation in the hippocampus of HFD mice. The reduction of brain insulin sensitivity is one of the most well-known causes of HFD-induced cognitive decline. Thus, we tested the hypothesis that insulin resistance could favor nutrient depletion and that AMPK-mediated inhibition of GFAT1 mice might be associated with the development of brain insulin resistance.

In detail, we observed an increase of IRS-1 phosphorylation on its inhibitory sites (Ser307 and 636) [66,67] in the hippocampus of HFD mice in comparison to standard-fed animals (Figure 3F,G; ** *p* < 0.01, SD vs. HFD: +108% and +70%). Furthermore, a substantial increase in p^Ser473^Akt levels was detected in the hippocampus of the model examined compared to the control group (Figure 3F,H; ** *p* < 0.01, SD vs. HFD: +73%) without affecting Akt protein expression levels between the two groups (Figure 3F,H). These data show an alteration of the insulin signaling pathway, characterized by the uncoupling between IRS1 and Akt, in agreement with previous studies [6,14,66,67].

### 2.3. Alzheimer’s Disease Hallmarks in HFD Mouse Brain

**Figure 4:**
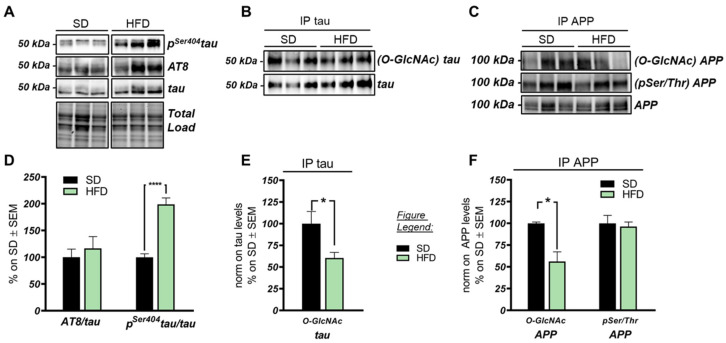
Role of altered protein O-GlcNAcylation in the onset of Alzheimer’s disease (AD) hallmarks in the brain of HFD mice. (**A,D**): Analysis of tau phosphorylation status in HFD hippocampus compared to SD-fed animals. A trend of increase was observed in ^Ser202-Thr205^tau/tau (AT8), together with a significant increase in ^Ser404^tau/tau that was observed in HFD mice compared to respective controls. Representative blots are reported in (**A**). (**B**,**E**) Evaluation of ^O-GlcNAc^tau levels by immunoprecipitation analysis. ^O-GlcNAc^tau/tau levels were remarkably reduced in the hippocampi of HFD mice in comparison to SD mice. Representative blots are reported in (**B**). (**C**,**F**) Evaluation of ^O-GlcNAc^APP and ^pSer/Thr^APP levels by immunoprecipitation analysis. ^O-GlcNAc^APP/APP was significantly reduced in HFD mice in comparison to SD animals, not supported by an inverse increase in its phosphorylated levels. Representative blots are reported in (**C**). Number of animals for each condition were as follow: *n* = 6/group for Western blot analysis, while *n* = 3/group was used for immunoprecipitation analysis. All bar charts reported in (**D**) and (**E**,**F**) show mean ± SEM. * *p* < 0.05, **** *p* < 0.0001 using Student’s t test.

A study by Kothari et al. recently proved that mice fed with a hypercaloric diet developed increased amyloid beta deposition and tau phosphorylation, which are associated with cognitive decline [68]. Considering this evidence, we evaluated tau and APP PTMs to assess the role of reduced O-GlcNAcylation in the development of these pathological hallmarks. At first, we confirmed that tau was hyperphosphorylated in the hippocampus of our HFD mice, which showed the increase in Ser404 phosphorylation in HFD mice compared to respective SD-animals (Figure 4D; **** *p* < 0.0001, SD vs. HFD: +98%). Furthermore, as observed by others [69], tau protein levels were increased by HFD (Figure S1; *p* = 0.05, SD vs. HFD: +44%).

The analysis of tau PTMs through immunoprecipitation highlighted the inverse relationship between O-GlcNAcylation and phosphorylation, at least for Ser404. Indeed, the p^Ser404^tau increased in HFD mice, meanwhile their O-GlcNAcylated levels significantly decreased when compared to SD animals (Figure 4B,E; * *p* < 0.05, SD vs. HFD: −37%). Considering APP PTMs, we also observed a significant reduction of ^O-GlcNAc^APP levels in the brain of HFD mice compared to SD levels (Figure 4C,F; * *p* < 0.05, SD vs. HFD: −56%), which was not supported by an inverse increase in its phosphorylation state (Figure 4C,F). In line with this result, we did not find an increased production of soluble Aβ 1–42 in HFD compared to SD animals (Figure S3). According to our results, the impairment of protein O-GlcNAcylation observed in the hippocampus of HFD mice might contribute to tau toxicity by inducing an imbalance in the O-GlcNAcylation/phosphorylation ratio of the protein, however we did not observe similar implications for APP.

### 2.4. High-Fat-Diet Affects Mitochondrial Function

**Figure 5:**
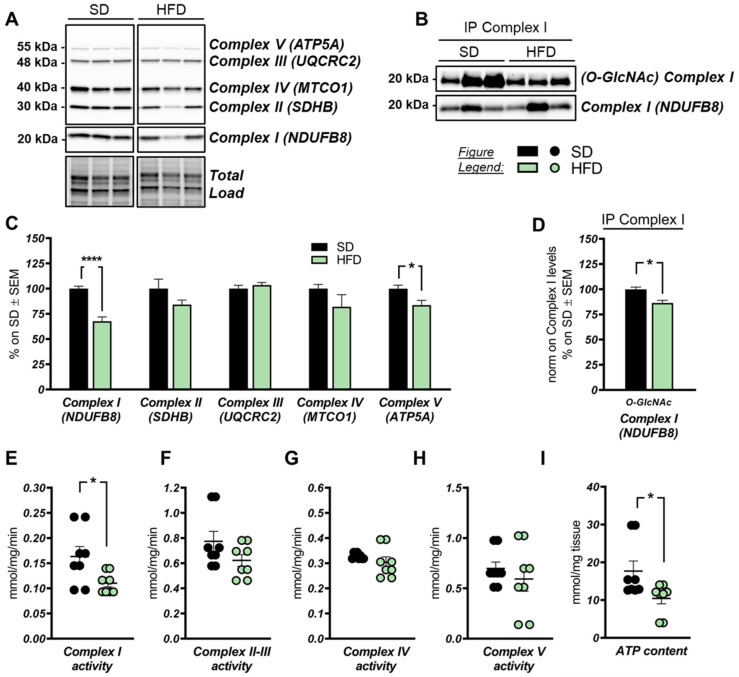
A high-fat diet affects respiratory chain complex expression and mitochondrial functionality. (**A**,**C**) Analysis of respiratory chain complex subunit expression in HFD-fed mice hippocampi compared to SD-fed animals. A significant reduction in Complex I subunit NDUFB8 was observed in HFD mice compared to SD controls. A trend of impairment was found for Complex II subunit SDHB and Complex IV subunit MTCO1 between HFD and respective controls, while no relevant changes have been observed in Complex III subunit UQCRC2 between the two groups of comparison. Finally, a significant decrease of Complex V subunit ATP5A expression was found in HFD-fed mice hippocampi in comparison to respective SD mice. Representative blots are reported in (**A**). (**B**,**D**) Analysis of Complex I subunit NDUFB8 O-GlcNAcylated levels by immunoprecipitation in HFD mice compared to controls. A significant reduction in ^O-GlcNAc^NDUFB8 levels was observed in the hippocampus from HFD mice compared to respective SD-fed mice. Representative blots are reported in (**B**). (**E**–**H**) Evaluation of specific respiratory chain complex activity in HFD mice compared to controls. A significant reduction of Complex I activity was reported in HFD mice compared to SD animals (**E**). A trend of reduction was also observed in Complex II-III activity (**F**) and Complex V activity (**H**) in HFD mice compared to controls. I: Analysis of overall ATP content in HFD mice compared to correspondent SD animals. A relevant impairment of ATP content was observed in HFD animals compared to respective SD-fed mice. Number of animals for each condition were as follow: *n* = 6/group for Western blot analysis, *n* = 3/group for immunoprecipitation analysis, and *n* = 8/group for complex activity and ATP content evaluation. All bar charts reported in (**C**–**G**) and (**H**,**I**) show mean ± SEM. * *p* < 0.05 using Student’s t test.

Mitochondrial dysfunction and impaired mitochondrial dynamics are known to be involved in the development of AD [70]. Studies on HFD-fed rats demonstrated the occurrence of abnormal mitochondrial density and morphology, together with altered mitochondrial dynamics, reduced respiratory chain complexes activity, and increased AMP/ATP ratio in myocardial tissue [71]. According to our analysis, HFD consumption induced an impairment in the expression levels of most respiratory chain complexes in comparison to that of SD mice. In detail, a consistent reduction of Complex I subunit NDUFB8 was observed in the hippocampus of HFD mice (Figure 5A,C; **** *p* < 0.0001, SD vs. HFD: −32%) and a similar but not significant trend of reduction was observed for Complex II subunit SDHB and Complex IV subunit MTCO1. No alteration was observed for Complex III subunit UQCRC2 between the two groups (Figure 5A,C). In addition, a significant reduction of Complex V subunit ATP5A was observed in the hippocampal region of HFD mice (Figure 5A,C; * *p* < 0.05, SD vs. HFD: −16%). The reduced expression of specific respiratory chain complex subunits was in line with data on mitochondrial complex activities. Indeed, a significant reduction of Complex I activity and a significant reduction of ATP content was detected in HFD mice compared to the respective control group (Figure 5E; * *p* < 0.05, SD vs. HFD: −0.053 mmol/mg/min; Figure 5I; * *p* < 0.05, SD vs. HFD: −7.22 mmol/mg tissue). To further understand the association between reduced protein O-GlcNAcylation and impaired mitochondrial function, we performed an immunoprecipitation analysis on Complex I subunit NDUFB8, whose function is regulated by O-GlcNAc levels [46,47,72]. As expected, we found a relevant reduction of NDUFB8 O-GlcNAcylated levels in HFD mice hippocampi compared to the SD group (Figure 5B,D; * *p* < 0.05, SD vs. HFD: −14%), supporting the idea that alteration of protein O-GlcNAcylation levels may cause of mitochondrial dys-function in HFD mice.

### 2.5. High-Fat-Diet Does Not Alter the Expression of Synaptic Proteins

**Figure 6:**
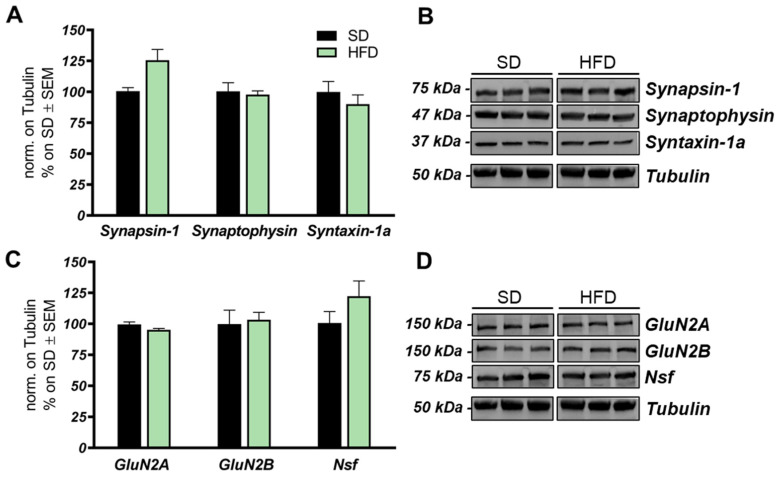
Expression levels of pre- and postsynaptic proteins in HFD-fed mice hippocampi. (**A**,**B**) Bar graph and Western blot images of synapsin-1, synaptophysin, and synthaxin-1a in the hippocampus of HFD mice compared to SD mice. (**C**,**D**) Bar graph and Western blot images of GluN2A and GluN2B subunits of glutamate NMDA receptor and NSF in the hippocampi of HFD mice compared to SD mice. Number of animals for Western blot analysis was 6/group. All bar charts reported in A and C show mean ± SEM. No significance was observed using Student’s t test.

Metabolic stress and alteration of mitochondrial function in brain areas may affect synaptic activity and induce synapse loss [73]. To evaluate the effects of HFD regimen on the expression of synaptic proteins, we analyzed the levels of pre- and postsynaptic markers in the hippocampi of HFD-fed mice. Immunoblot analysis did not detect any change in the expression of synaptophysin, synapsin-1, soluble N-ethylmaleimide-sensitive fusion protein attachment protein receptors (SNARE) complex proteins syntaxin-1a and NSF, and glutammate N-methyl-D-aspartate (NMDA) receptor subunits GluN2A and GluN2B (Figure 6A–D).

### 2.6. Insulin and Palmitic Acid (IPA) Treatment in SHSY-5Y Neuroblasoma Cells Ties Insulin Resistance with Reduced O-GlcNAcylation and Mitochondrial Defects

**Figure 7:**
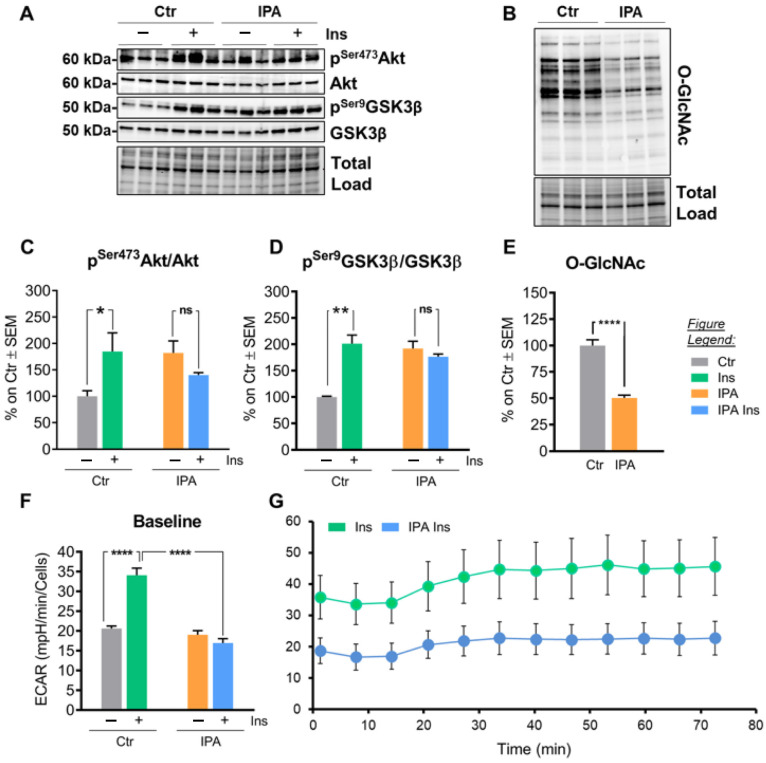
Insulin and palmitic acid (IPA) treatment in SHSY-5Y neuroblastoma cells lead to insulin resistance and reduced protein O-GlcNAcylation. (**A,C**): Analysis of Akt activation status in IPA-treated cells under the stimulus of insulin. Control cells rapidly responded to the insulin stimulus by phosphorylating Akt on Ser473, while IPA-treated cells did not show relevant changes in pSer473Akt/Akt ratio under the stimulus of insulin. Representative blots are reported in (**A**). (**A**,**D**) Analysis of GSK3β activation status in IPA-treated cells under the stimulus of insulin. GSK3β was phosphorylated on Ser9 by Akt in control cells treated with insulin, while IPA pretreated cells were not responsive. Representative blots are reported in (**A**). (**B**,**E**) Evaluation of global O-GlcNAcylated proteins in IPA-treated cells compared to controls. IPA treatment induced a significant reduction in the overall levels of O-GlcNAcylated proteins. Representative blot is reported in (**B**). (**F**,**G**) Bioenergetic profile of neuroblastoma cells after insulin treatment. Representative blot is reported in (**B**). (**F**,**G**) Bioenergetic profile of neuroblastoma cells after insulin treatment. The glycolytic flux, obtained by measuring extracellular acidification rate (ECAR) by Seahorse experiments, of +/− IPA pretreated neuroblastoma cells after +/− 30′ of insulin incubation. In (**F**) the basal average value of +/− IPA treated and +/− insulin stimulated cells is shown; in (**G**) the time-course of the insulin sample +/− IPA pretreatment is shown. The time-course of non-stimulated cells is shown in Figure S2. A sample experiment is shown. Number of replicates for each condition were as follow: *n* = 6/group for Western blot analysis and *n* = 16/group for Seahorse experiment. All bar charts reported in (**C**,**D**) and (**E**,**F**) show mean ± SEM. Bar charts reported in (**G**) show mean ± SD. * *p* < 0.05, ** *p* < 0.01, **** *p* < 0.0001, ns = not significant using Student’s t test and 1-way ANOVA.

Subsequently, we further investigated the relationship between HFD-induced metabolic alterations and impaired protein O-GlcNAcylation by adapting an in vitro model previously developed by Spinelli et al. [14]. Briefly, SHSY-5Y neuroblastoma cells were pretreated for 24 h with a mixture of insulin and palmitic acid (IPA) in order to reproduce the metabolic changes observed in HFD mice hippocampi. Since HFD consumption has been widely associated with the occurrence of brain insulin resistance [68,74], we firstly tested the ability of pretreated IPA protocol to alter the insulin cascade in cells. Under normal conditions, control cells rapidly respond to the insulin stimulation by phosphorylating Akt on Ser473 (Figure 7A,C; * *p* < 0.05, CTR vs. Ins: +85%), which in turn inhibits GSK3β trough phosphorylation on Ser9 (Figure 7A,D; ** *p* < 0.01, CTR vs. Ins: +101%). As expected, SHSY-5Y pretreated with IPA were unable to respond to the insulin pulse, as revealed by the unchanged levels of p^Ser473^Akt/Akt (Figure 7A,C; n.s. IPA vs. IPA Ins) and p^Ser9^GSK3β/GSK3β (Figure 7A,D; n.s. IPA vs. IPA Ins), confirming the occurrence of insulin resistance, as previously observed [14]. To further analyze the effect of IPA on metabolism, the energetic profiles of neuroblastoma cells were assessed through the Cell Mito Stress Test using the extracellular flux analyzer XFe96 (Seahorse, Agilent). The glycolytic flux (obtained as extracellular acidification rate, ECAR) increased after 30′ insulin pulse in CTR but not in IPA-pretreated SHSY-5Y cells, supporting the impairment of glycolytic machinery as an effect of insulin resistance (Figure 7F,G; **** *p* < 0.0001, CTR vs. Ins: +75%; **** *p* < 0.0001, Ins vs. IPA Ins: −80%).

**Figure 8:**
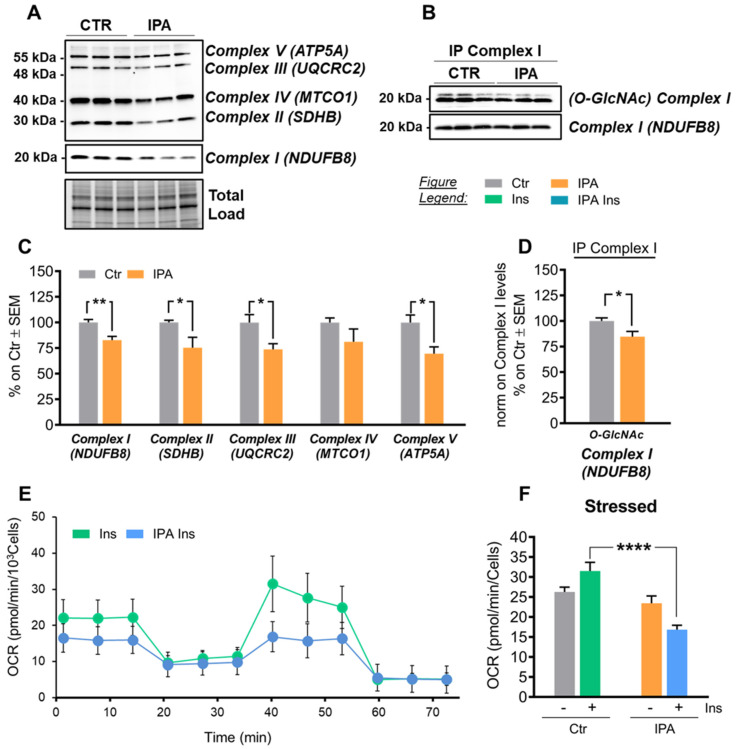
IPA treatment in SHSY-5Y neuroblastoma cells promotes mitochondrial dysfunction. (**A**,**C**) Analysis of respiratory chain complex subunit expression in IPA-treated cells compared to controls. IPA treatment induced a consistent impairment in the protein expression levels of roughly all the mitochondrial complexes: Complex I (subunit NDUFB8), Complex II (subunit SDHB), Complex III (subunit UQCRC2), and Complex V (ATP5A). A trend of reduction was also observed in Complex IV (MTCO1) protein expression levels. Representative blots are reported in (**A**). (**B**,**C**) Immunoprecipitation analysis of the O-GlcNAcylated levels of Complex I subunit NDUFB8 in IPA-treated cells in comparison to controls. IPA-treated cells showed a significant impairment in NDUFB8 O-GlcNAcylated levels. Representative blots are reported in (**B**). (**E**,**F**) Bioenergetic profile of neuroblastoma cells after insulin treatment. (**E**) The mitochondrial respiration of +/− IPA pretreated neuroblastoma after 30′ of insulin incubation, obtained by means of OCR by Seahorse experiments. (**F**) The stressed OCR graph comparing +/− IPA-treated cells subjected or not to insulin incubation. In the figure, a representative experiment, values reported in the plot are the means of 16 replicates ± SD. Time course of the control samples, for both OCR and ECAR are reported in Figure S2. Number of replicates for each condition were as follow: *n* = 6/group for Western blot analysis and *n* = 16/group for Seahorse. All bar charts reported in (**C**) and (**D**,**F**) show mean ± SEM. Bar charts reported in (**E**) show mean ± SD. * *p* < 0.05, ** *p* < 0.01, **** *p* > 0.0001 using Student’s t test and 1-way ANOVA.

Interestingly, in IPA cells, the reduced capability to respond to insulin was associated with a significant reduction of total O-GlcNAcylated proteins (Figure 7B,E; **** *p* < 0.0001, CTR vs. IPA: −50%), reproducing the alterations observed in HFD-fed mice hippocampi. These data confirm a role for protein O-GlcNAcylation as a sensor of neuronal metabolism. According to our analysis, IPA treatment also induced a reduction of the expression of protein belonging to the respiratory chain complexes. In detail, a significant decrease of Complex I subunit NDUFB8 protein levels was observed in IPA-treated cells (Figure 8A,C; ** *p* < 0.01, CTR vs. IPA: −18%), as well as a relevant reduction of Complex II subunit SDHB levels (Figure 8A,C; * *p* < 0.05, CTR vs. IPA: −25%). In line with these results, Complex III subunit UQCRC2 (Figure 8A,C; * *p* < 0.05, CTR vs. IPA: −26%) and Complex V subunit ATP5A protein levels (Figure 8A,C; * *p* < 0.05, CTR vs. IPA: −30%) were also decreased in IPA-treated cells in comparison to controls. Furthermore, the immunoprecipitation analysis of Complex I subunit NDUFB8 demonstrated the reduction of O-GlcNAcylated levels (Figure 8B,D; * *p* < 0.05, CTR vs. IPA: −15%) after IPA treatment, as previously observed in HFD mice, confirming the strong correlation between insulin resistance-induced mitochondrial defects and impaired O-GlcNAcylation profile.

The bioenergetic metabolism was also measured to evaluate the oxygen consumption rate (OCR) under both basal and stressed conditions (Figure 8E,F); to promote stressed conditions specific drugs were used to target (according to the addition order): (i) complex V, i.e., ATP production; (ii) membrane potential, i.e., by uncoupling electron transfer and proton translocation; (iii) electron transfer chain, i.e., complex I and III. The analysis of OCR under stressed condition demonstrated that insulin stimulation of IPA-treated cells negatively affects the maximal respiratory capacity of mitochondria (Figure 8E,F, **** *p* < 0.0001 Ins vs. IPA Ins: −40%), indicating that the metabolic potential of such cells is negatively affected by the development of insulin resistance.

## 3. Discussion

**Figure 9:**
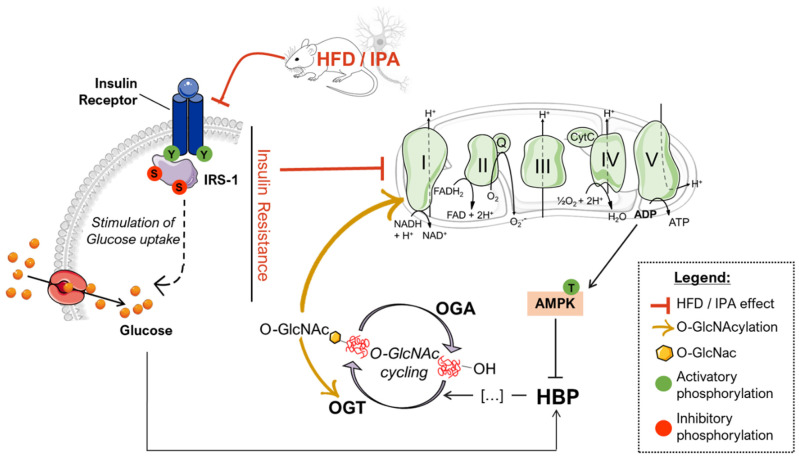
Proposed scenario of the altered mechanisms driven by HFD/IPA treatment. According to our data, nutrient overload results in defective insulin signaling both in HFD mice and IPA-treated SHSY-5Y cells, which translate into reduced glycolytic and mitochondrial metabolism. In turn, mitochondrial defects are responsible for impaired bioenergetic and increased ADP/ATP ratios that activate AMPK, a key GFAT1 inhibitor, leading to the reduced entrance of nutrients into the hexosamine biosynthetic pathway (HBP). GFAT1 inhibition, together with insulin resistance-driven nutrient deprivation, is responsible for the impairment of the HBP flux and the consequent reduction of the overall levels of O-GlcNAcylated proteins. The disruption of O-GlcNAc homeostasis is associated with altered OGT PTMs that could further exacerbate the efficiency of O-GlcNAc cycling. In addition, the reduction of Complex I-specific O-GlcNAcylation may represent a key aspect involved in exacerbating the alterations mitochondrial energetic profile.

Studies on the high-fat-diet (HFD) model have largely demonstrated a strong correlation between the consumption of an hypercaloric diet and consequent alterations in brain functionality [75,76,77,78]. However, the exact molecular mechanisms that link perturbations of peripheral metabolism with brain alterations driving cognitive decline are still under discussion. Protein O-GlcNAcylation is a post-translational modification of quite recent discovery that is extremely sensitive to nutrient fluctuations, thus it has been widely recognized as a key linkage between nutrient sensing, energy metabolism, and cellular functions [79,80]. Several lines of evidence have emphasized the role of aberrantly increased protein O-GlcNAcylation in driving glucose toxicity and chronic hyperglycemia-induced insulin resistance [56,60], major hallmarks of T2DM and obesity. In this regard, the HFD model closely recapitulates molecular changes occurring in the so-called metabolic syndrome, which manifests as hyperglycemia, hyperinsulinemia, and insulin resistance and precedes obesity and T2DM [52,81,82]. Our analysis of liver samples from HFD mice summarized the increase in protein O-GlcNAcylation already observed in the peripheral organs of diabetic individuals, confirming O-GlcNAcylation as a major trigger of glucose toxicity [56,83,84]. A completely different scenario is to be considered for the role of O-GlcNAcylation in the brain. A wide range of evidence has already demonstrated how a hypercaloric diet affects cognitive performances, promoting metabolic dysregulation in the brain in a way similar to diabetes [75,81,85,86]. In agreement, epidemiological and molecular studies have suggested that T2DM, obesity, and general defects in brain glucose metabolism predispose to poorer cognitive performance and rapid cognitive decline during ageing, favoring the onset of dementia [40,87,88]. In this scenario, the alteration of protein O-GlcNAcylation that we observed in the hippocampus from HFD mice, coupled with the aberrant increase in Ser/Thr phosphorylation levels, resemble the alterations observed in the brain of AD individuals [31,36,89,90,91], as well as, of AD and DS mouse models. Indeed, previous studies have demonstrated the reduction of total protein O-GlcNAc levels in the brain from AD and DS mice, supporting its involvement in the early molecular mechanisms promoting the development of AD signatures [22,55,92]. In line with these reports, the analysis of rat brains fed a short-term HFD demonstrated the increase of Aβ deposition and p-tau, and decreased synaptic plasticity, suggesting that molecular mechanism underling the onset of cognitive decline in HFD mice may resemble those occurring in AD models [93]. Our current investigation of tau and APP PTMs show that the alteration of their O-GlcNAcylation/phosphorylation ratio may represent one of the molecular mechanisms involved in HFD-related neurodegeneration. In particular, the significant reduction of tau O-GlcNAcylation levels, associated with the pathological increase in Ser404 phosphorylation, suggest a contrition of reduced O-GlcNAc levels in promoting tau toxicity [94,95]. As well, decreased O-GlcNAc levels of APP was shown to favor amyloidogenic processing and Aβ deposition through the increase of APP phosphorylation [96,97], however no altered levels of APP phosphorylation were observed in our study, as well as no accumulation of soluble Aβ 1–42. Although most of the pathological alterations observed in HFD mice are similar to those of other neurodegeneration models, the mechanisms underlying disturbances of O-GlcNAc homeostasis in the brain of HFD mice have different origins. We previously observed in the hippocampus of a mouse model of DS, the hyperactivation of OGA [55], but HFD mice did not show any significant increase in the removal of O-GlcNAc moiety. Rather the attention must be focused on the observed alteration of the hippocampal metabolic status associated with the development of insulin resistance. Previous human and rodent data support diet-induced insulin resistance as one of the main mediators of cognitive deficit associated with high fat consumption [68,82,93]. In agreement, mice fed with a HFD exhibited a significant increase in obesity and lower glucose and insulin tolerance as compared to animals fed with a standard diet. These changes parallel the consistent alterations of the insulin signaling in the brain characterized by increased IRS1 inhibition and the uncoupling with Akt [68,82]. These molecular events are key features of brain insulin resistance [6,66,67,74] (Figure 9). Indeed, as previously reported in HFD-treated mice, hyperactive Akt fails to further respond to insulin administration because of IRS-1 inhibition [14]. Interestingly, inhibitory serine residues of IRS1 can also be O-GlcNAcylated [22,98] leading to the possibility of reduced O-GlcNAcylation as one of the leading causes for IRS-1 hyperphosphorylation on inhibitory sites. Although it seems clear that the disruption of the balance between O-GlcNAcylation and phosphorylation contributes to the unproper functioning of the insulin cascade, it should also be considered that the onset of insulin resistance might affect O-GlcNAc homeostasis by altering the number of metabolites available for the synthesis of UDP-GlcNAc. In line with a metabolic profile characterized by low nutrient availability and increased AMP/ATP ratio, HFD mice showed a significant increase in AMPK activation (increased ^pThr172^AMPK levels). Most interestingly, AMPK-mediated inhibition of GFAT1 activity by its direct phosphorylation on Ser243 appears as one of the best-characterized mechanisms to reduce the amount of metabolites entering the HBP flux under nutrient depletion conditions [62,63,64]**,** matching the changes observed in HFD mice brains (Figure 9). OGT is known to be phosphorylated by AMPK on Thr444, regulating both OGT selectivity and nuclear localization. Furthermore, the regulation of OGT expression and O-GlcNAc levels by AMPK seems to be highly dependent on cell type and pathological status, and glucose-deprivation was proven to favor OGT cytosolic localization upon AMPK activation [58,99,100,101]. Within this context, AMPK subunits have been shown to be dynamically modified by O-GlcNAc, which regulates their activation, showing a complex interplay between these enzymes [99]. Most interestingly, OGT O-GlcNAcylation/phosphorylation ratio was unbalanced in the hippocampus of HFD mice, suggesting the alteration of OGT function. Besides, the observed reduction of OGT phosphorylation on Tyr residues further supports the impairment of its activity, as suggested by Hart et al. [59]. According to this evidence, HFD-driven alterations of protein O-GlcNAcylation seems to be linked to reduced HBP flux and altered OGT PTMs, which contributes to aberrant O-GlcNAc cycling. In parallel to insulin resistance, HFD rodents also show altered mitochondrial functionality, oxidative stress, and reduced brain cortex bioenergetic [71,77,102]. In agreement, among the possible mechanisms by which nutrient overload exerts negative effects within the brain, the alteration of mitochondrial functionalities is among the most impactful. In a recent study, Chen et al. have shown an impaired expression of protein involved in mitochondrial dynamics, reduced Complex I–III activity and decreased mitochondrial respiration in HFD-treated rats [71]. Furthermore, a reduced AMP/ATP ratio in HFD rats demonstrated how diet-induced impairment of mitochondrial activity is reflected by the reduced ability to synthetize ATP as a source of energy [71]. In this scenario, data collected in the hippocampus of HFD mice are in line with recent studies on the topic, supporting that high fat consumption can affect mitochondria functionality, thus resulting in reduced respiratory capacity, decreased oxygen consumption, and reduced ATP production [77,102]. Indeed, we demonstrated in HFD mice, a significant reduction of both the expression levels and the activity of most respiratory chain complexes, especially Complex I, finally resulting in impaired ATP content in comparison to SD mice (Figure 9).

Within this context, sustained alterations in O-GlcNAcylation either by pharmacological or genetic manipulation are known to reprogram mitochondrial function and may underlie reduced cellular respiration and ROS generation [103]. Furthermore, marked reduction of global O-GlcNAcylation strongly correlates with hampered bioenergetic function and disrupted mitochondrial network in AD models, while TMG-mediated restoration of overall O-GlcNAcylation has shown neuroprotective effects [36]. Mitochondria dysfunction is a hallmark of physiological and pathological brain ageing, which may prelude to impairment of synaptic activity and depletion of synapse. However, six-week HFD protocol did not affect the expression of pre- and postsynaptic proteins, suggesting that alteration of nutrient-dependent signaling and metabolic activity in neurons may anticipate the synaptic plasticity and structural plasticity deficits observed in experimental models of metabolic disease [104,105]. Intriguingly, we also highlighted that the impairment of the Complex I could be associated with the reduced O-GlcNAcylated levels of its subunit NDUFB8, as described elsewhere [46,47,72], thus describing a pivotal role for altered protein O-GlcNAcylation in triggering mitochondrial defects mediated by high-fat consumption (Figure 9).

In agreement with data collected in HFD, the administration of IPA for 24 h to SHSY-5Y neuroblastoma cells was able to induce insulin resistance, as previously observed in primary neurons [14], which translated into the lowered activity or readaptation of the glycolytic machinery and into the reduced mitochondrial respiratory potential in response to insulin stimulation. Intriguingly, as observed in HFD mice, IPA-induced insulin resistance and metabolic changes, after IPA treatment, were associated and self-sustained by total and Complex I-specific reduction of protein O-GlcNAcylation in neuronal-like cells, confirming the crucial role of such PTMs as sensor and mediator of energetic fluctuations.

Overall, our data suggest that a diet rich in fat is associated with insulin resistance and altered metabolism of the brain, which translates into reduced protein O-GlcNAcylation and mitochondrial dysfunction. In particular, HFD-related aberrant protein O-GlcNAcylation seems to have a double role in promoting neurodegeneration, by triggering the hyperphosphorylation of tau and by reducing complex I activity.

## 4. Materials and Methods

### 4.1. Animal Model

Male C57BL/6 mice (30–35 days old), derived from Animal Facility of Università Cattolica del Sacro Cuore, were randomly assigned to two feeding regimens: (I) standard diet (SD, control) and (II) high-fat diet (HFD, whose caloric intake was composed by 60% of saturated fatty acids). Animals were housed under a 12-h light–dark cycle at room temperature (RT: 19–22 °C) and fed with their respective diet (Mucedola Srl, Settimo Milanese, Italy) and water ad libitum. After 6 weeks of the nutritional regimen, animals were sacrificed and tissues (hippocampus and liver) were collected, flash-frozen, and stored at −80° C until utilization. All animal procedures were approved by the Ethics Committee of Università Cattolica del Sacro Cuore, experimental protocol was approved by Italian Ministry of Health (#39/2017-PR, approved on 16 January 2017) and were fully compliant with Italian (Ministry of Health guidelines, Legislative Decree No. 116/1992, 27 January 1992) and European Union (Directive No. 210/63/EU, 22 September 2010) legislations on animal research. The methods were carried out in strict accordance with the approved guidelines.

### 4.2. IPA Treatment in Neuroblastoma Cells

SHSY-5Y cells were grown in Dulbecco’s modified Eagle’s medium: Nutrient Mixture F12 (DMEM/F12; Aurogene, Rome, Italy), supplemented with 10% fetal bovine serum (FBS; Aurogene) and 1% penicillin (Sigma-Aldrich, St. Louis, MO, USA). Cells were maintained at 37 °C in a saturated humidity atmosphere containing 95% air and 5% CO_2_. SHSY-5Y were seeded in 24-wells plates (150 k/well) and 24-h before the treatment cells were switched from 10% to 1% FBS-containing medium. In order to mimic the effects of high fat diet, cells were exposed to a mixture of palmitic acid (200 µM; P0500, Sigma-Aldrich) and insulin (20 nM; 91077C, Sigma-Aldrich) for 24 h, and eventually stimulated for 30 min with insulin (100 nM; Humulin R, Ely-Lilly, Inadianapolis, IN, USA). Cells were then washed twice with PBS, collected, and proteins were extracted as described below.

### 4.3. Sample Preparation for Western Blot and Immunoprecipitation

In order to obtain total protein extracts, tissues and cells were homogenized in RIPA buffer (pH 7.4) containing 50 mM Tris-HCl (pH 7.4), 150 mM NaCl, 1% NP-40, 0.25% sodium deoxycholate, 0,1% SDS, 1 mM EDTA, protease inhibitor cocktail (1:100; 539132, Millipore), phosphatase inhibitor cocktail (1:100; P5726, Sigma-Aldrich), Benzyl-2-Acetamido-2-Galactopyranose (OGT inhibitor, 2 mM; B4894, Sigma-Aldrich), and PUGNAc (OGA inhibitor, 100 μM; A7229, Sigma-Aldrich). As an additional step, hippocampus and liver samples from SD and HFD mice were homogenized by 20 strokes of a Wheaton tissue homogenizer before clarification. Sample homogenates were then sonicated and centrifuged at 14,000 rpm for 40 min at 4 °C to remove debris. Supernatant was collected and the BCA method (Pierce™ BCA Protein Assay Kit, 23,227, ThermoFisher Scientific, Waltham, MA, USA) was used according to manufacturer instructions to determine total protein concentration.

### 4.4. Western Blot

For Western blot analysis, 15 μg of proteins were separated via SDS-PAGE using Criterion™ TGX Stain-Free™ precast gel (Bio-Rad, Hercules, CA, USA) and transferred to a nitrocellulose membrane by Trans-Blot Turbo Transfer System (Bio-Rad). The blot was imaged by ChemiDoc MP imaging system (Bio-Rad) using the Stain-Free Blot settings. Protein total load captured by Stain-Free technology was later used for total protein normalization. Following this, nitrocellulose membrane was blocked using 3% BSA (bovine serum albumin; 9048-46-8, SERVA, Heidelberg, Germany) or milk 5% (skim milk powder; 42590, SERVA) in 1X Tris buffer saline (TBS; #1706435, Bio-Rad) containing 0.01% Tween20 and incubated overnight at 4 °C with the following primary antibodies: pThr172AMPK (1:1000; GTX52341, GeneTex, Irvine, CA, USA), AMPKα1/2 (1:500; SC-74461, Santa Cruz Biotechnology, Dallas, TX, USA), pSer473Akt (1:1000; 4058S, Cell Signaling Technology, Danvers, MA, USA), Akt (1:1000; VMA00253, Bio-Rad Laboratories), APP (1:5000; SAB5200113, Sigma-Aldrich), AT8 (1:1000; MN1020, TermoFisher Scientific, Waltham, MA, USA), pSer243GFAT1 (1:1000; S343C, MRC-PPU, Dundee, United Kingdom), Complex I (NDUFB8, 1:1000; NBP2-75586, Novus Biological, Centennial, CO, USA), GFAT1 (1:1000; 28121, IBL-America, Minneapolis, MN, USA), pSer307IRS1 (1:1000; 2381S, Cell Signaling Technology), pSer636IRS1 (1:1000; GTX32400, GeneTex), IRS-1 (1:500; SC-8038, Santa Cruz Biotechnology), OGA (1:1000; SAB-4200267, Sigma-Aldrich), O-GlcNAc CTD110.6 (1:500; SC-59623, Santa Cruz Biotechnology), O-GlcNAc RL2 (1:1000; MABS157, Sigma-Aldrich), OGT (1:500; SC-74546 Santa Cruz Biotechnology), pSer/Thr (1:5000; ab17464, Abcam, Cambridge, United Kingdom), pSer404tau (1:1000; ab92676, Abcam), tau (1:1000; orb46243, Biorybt, St Louis, MO, USA), total OXPHOS (1:3000; Ab110411, Abcam), synaptophysin (1:1000; Ab8049, Abcam), synapsin-1 (1:1000, #5297, Cell Signaling Technology), syntaxin-1a (1:1000, Ab41453, Abcam), NSF (1:1000, #3924, Cell Signaling Technology), GluN2B (1:1000, 610417, BD Biosciences, San Jose, CA, USA), GluN2A (1:1000, 07-632, Millipore, Burlington, MA, USA), and Tubulin (1:1000, 16074, Sigma). The next day, all membranes were washed with 1X TBS containing 0.01% Tween20 and incubated at RT for 1 h with respective horseradish peroxidase-conjugated secondary antibodies: anti-rabbit (1:10000; L005661, Bio-Rad Laboratories), anti-mouse (1:10000; L005662, Bio-Rad Laboratories), and anti-sheep, (1:3000; A3415, Sigma-Aldrich). As necessary, enhanced sensitivity was obtained using secondary antibodies able to detect only native IgG (1:200; TidyBlot, #STAR209, Bio-Rad Laboratories. 1:1000; TrueBlot, 18-8817-30, Rockland Immunochemicals, Pottstown, PA, USA). Blots were then imaged via the ChemiDoc MP imaging system using chemiluminescence settings. Subsequent determination of relative abundance via total protein normalization was calculated using Image Lab 6.1 software (Bio-Rad Laboratories).

### 4.5. Immunoprecipitation

For OGT: Sepharose beads were used to immunoprecipitate OGT (EZView Red Protein G Beads, Sigma-Aldrich) according to manufacturer instructions. Briefly, different sample -sets (100 μg of proteins; *n* = 3/group) were incubated overnight at 4 °C with the primary antibody for OGT (1:100; SC-74546 Santa Cruz Biotechnology) in IP buffer containing 10 mM Tris (pH = 7.6), 140 mM NaCl, 0.5% NP40, phosphatase inhibitor cocktail (1:100; P5726, Sigma-Aldrich), PUGNAc (OGA inhibitor, 100 μM; A7229, Sigma-Aldrich), and benzyl-2-acetamido-2-galactopyranose (OGT inhibitor, 2 mM; B4894, Sigma-Aldrich). The next day, all samples were incubated with 20 μL of Protein G beads (EZView Red Protein G Beads, E3403, Sigma-Aldrich) for 2h at RT and then washed three times with RIA buffer containing 10 mM Tris (pH = 7.6), 140 mM NaCl, 1% NP40. Afterwards, standard Western blot procedure was performed. Resulting blots were incubated overnight at 4 °C with the following primary antibodies: O-GlcNAc CTD110.6 (1:500; SC-59623, Santa Cruz Biotechnology), O-GlcNAc RL2 (1:1000; MABS157, Sigma-Aldrich), OGT (1:500; SC-74546, Santa Cruz Biotechnology), pSer/Thr (1:5000; ab17464, Abcam), pTyr (1:5000; #9416, Cell Signaling Technology) and detected by the horseradish peroxidase-conjugated secondary antibodies, anti-mouse (1:10,000; L005662, Bio-Rad Laboratories) and anti-rabbit (1:10,000; L005661, Bio-Rad Laboratories). IP results were normalized on the total amount of OGT and analyzed following the same procedures used for Western blot.

For APP, tau and Complex I (NDUFB8): magnetic beads were used to immunoprecipitate APP, tau, and Complex I subunit (SureBeads™ Protein G Magnetic Beads; 1,614,023, Bio-Rad Laboratories) according to manufacturer instructions. Briefly, 100 μL of magnetic beads were magnetized using a specific tube magnetic rack and washed three times with 1X PBS containing 0.1% Tween20. Primary antibody for APP (1:100; SAB5200113, Sigma-Aldrich), tau (1:50; orb46243, Biorybt), or Complex I subunit NDUFB8 (1:75; NBP2-75586, Novus Biological) was incubated with magnetic beads for 30 min at RT. After three washes, 100 μg of proteins (*n* = 3/group) for each sample were incubated for 1 h and 30 min at RT. After an additional three washes, standard Western blot procedure was performed for APP, tau, and Complex I subunit NDUFB8 IP. Resulting blots were incubated overnight at 4 °C with the following primary antibodies: APP (1:5000; SAB5200113, Sigma-Aldrich), Complex I (NDUFB8, 1:1000; NBP2-75586, Novus Biological), tau (1:1000; orb46243, Biorybt), O-GlcNAc CTD110.6 (1:500; SC-59623, Santa Cruz Biotechnology), O-GlcNAc RL2 (1:1000; MABS157, Sigma-Aldrich), and pSer/Thr (1:5000; ab17464, Abcam) and detected by the horseradish peroxidase-conjugated secondary antibodies, anti-mouse (1:10,000; L005662, Bio-Rad Laboratories), anti-rabbit (1:10000; L005661, Bio-Rad Laboratories), and by horseradish peroxidase-conjugated secondary antibodies able to detect only native IgG (1:200; TidyBlot, #STAR209, Bio-Rad Laboratories; 1:1000; TrueBlot, 18-8817-30, Rockland Immunochemicals). IP results were normalized on the total amount of APP, tau, or Complex I subunit NDUFB8 and analyzed following the same procedures used for Western blot.

### 4.6. OGA Enzymatic Assay

OGA enzymatic activity was measured using the synthetic substrate p-nitrophenyl N-acetyl-β-D-glucosaminide (pNP-GlcNAc) as described by Zachara et al. [106]. Briefly, ~15 mg of hippocampus from SD and HFD mice (*n* = 6/group) were thawed in RIPA buffer (pH = 7.4) containing 50 mM Tris (pH = 7.4), 50 mM NaCl, 1% NP-40, 0.25% sodium deoxycholate,1 mM EDTA, 0,1% SDS, protease inhibitor cocktail (1:100; 539,132, Millipore), and phosphatase inhibitor cocktail (1:100; P5726, Sigma-Aldrich). Brains were homogenized by 20 strokes of a Wheaton tissue homogenizer, sonicated, and centrifuged at 14 000 rpm for 40 min at 4 °C to remove debris. Supernatant was collected, desalted using Zeba™ Spin Desalting Columns (89,882; ThermoFisher Scientific), and protein concentration was determined by the BCA method (Pierce™ BCA Protein Assay Kit, 23227, ThermoFisher Scientific) according to manufacturer instructions. Samples (150 μg of proteins) were incubated with activity assay buffer containing 2 mM pNP-GlcNAc, 50 mM sodium cacodylate (pH = 6.4), 50 mM N-acetylgalactosamine, and 0.3% BSA at 37 °C for 2 h. Reaction was stopped by the addition of 500 mM Na2CO3 and absorbance was measured at 405 nm (Multiskan EX, Thermo Labsystems). OGA activity was reported as enzyme activity units, where 1U catalyzed the release of 1 μmol pNP/min from pNP-GlcNAc.

### 4.7. GFAT1 Enzymatic Assay

GFAT1 enzymatic activity was performed adapting a procedure developed by McClain and colleagues [65]. GFAT1 activity for HFD and SD mice was assessed though the measuring of its enzymatic product glucosamine 6-phosphate (GlcN6P). Briefly, ~15 mg of hippocampus (*n* = 6/group) were thawed in 80 μL Lysis buffer (pH = 7.5) containing 100 mM KCl, 1 mM EDTA, 50 mM Na3PO4, protease inhibitor cocktail (1:100; 539,132, Millipore), and phosphatase inhibitor cocktail (1:100; P5726, Sigma-Aldrich). Brains were homogenized by 20 strokes of a Wheaton tissue homogenizer, sonicated, and centrifuged at 14,000× *g* for 40 min at 4 °C to remove debris. Supernatant was collected and total protein concentration was determined by the BCA method (Pierce™ BCA Protein Assay Kit, 23,227, Thermo Fisher Scientific) according to manufacturer instructions. Samples (240 μg of proteins) were incubated with activity assay buffer containing 1 mM EDTA, 1 mM DTT, 40 mM NaHPO4 (pH = 7.4), and 12 mM fructose 6-phosphate and 12 mM L-glutamine at 37 °C for 45 min. Reaction was stopped by the addition of PCA 1 M (1:2) to induce protein precipitation. Samples were then incubated 10 min on ice and centrifuged at 16,000× *g* for 10 min at 4 °C. Supernatant was extracted with chloroform (1:2) and 100 μL of the aqueous phase was collected for HPLC analysis. GlcN6P generated during the reaction was detected by derivatization of the sample with 2-volumes of o-phthalaldehyde (OPA) reagent (100 μL of 10 mg/mL OPA in EtOH, 900 μL sodium borate 100 mM pH = 9.7 and 2 μL 3-mercaptopropionic acid). The reaction was incubated for 10 min at RT protected from light and sample was diluted 1:1000 in the mobile phase for HPLC detection. Chromatographic separation was performed using an isocratic elution, the mobile phase was composed by Na3PO4 15 mM, pH = 7.2 (phase A) and acetonitrile (phase B) (90:10). A Symmetry C18 column (300 Å, 5 μm, 4.6 mm × 250 mm, 1/pk, Waters Corporation) was used for separation. Fluorescence of the sample eluent (λ = 340/450) was analyzed using a fluorescent detector (RF-551, Shimadzu) and the peak area was integrated using a dedicated software (Empower 2, Waters Corporation). OPA-derivatized GlcN6P standards (G5509, Sigma-Aldrich) were run separately to determine the retention time (1.8 s) and to generate a standard curve to correlate area to activity. The correlation coefficient between the concentration of GlcN6P standards and the area under the GlcN6P peak was 0.999. Activity was expressed as U/mg protein where 1 U represented the generation of 1 pmol of GlcN6P/min.

### 4.8. Aβ 1-42 ELISA Analysis

Mouse Aβ 1-42 ELISA Kit (KMB3441; Invitrogen TermoFisher Scientific) was used to determine the levels of amyloid β 1–42 peptide in SD and HFD mice (*n* = 6/group). A comparable sample from a 3XTg model was used as a positive control (*n* = 2/group). Briefly, ~10 mg of hippocampus was thawed in ice-cold DEA buffer (10 µL/mg tissue; 0.2% Diethanolamine in 50 mM NaCl) with protease inhibitor cocktail (1:100; 539,132, Millipore). After centrifugation (15,000 rpm 1 h 30 min 4 °C), supernatant was retained as Aβ soluble fraction. Aβ 1-42 was then measured according to manufacturer’s instructions. Curve-fitting was obtained by Graph Pad Prism 8.0 software (GraphPad, La Jolla, CA, USA).

### 4.9. Mitochondria Isolation

Experiments were conducted on 4 samples per group. Due to the small amount of tissue available, each sample was obtained by pooling hippocampi from 2 animals (for a total of 8 mice per group). Mitochondria were isolated as previously described by using a gradient of Percoll [107]. Briefly, the hippocampi were weighted (100 mg) and immersed in ice-cold mitochondrial isolation buffer (MIB) containing 0.25 M sucrose, 0.5 mM K -EDTA, 10 mM Tris–HCl, pH 7.4. All the instruments and buffers were kept cold during the experiment. Each sample was homogenized in 3.8 mL of 12% Percoll in MIB (5% *w/v*) using Dounce homogenizers with glass pestles. A total of 3 mL of homogenate was then layered onto a previously poured, 3.5 mL 26% Percoll, on 3.5 mL 40% Percoll density gradient. The gradient was centrifuged at 19,000 rpm (30,000× *g*) in a Sorval RC-5B type rotor for 5 min. The resulting top layer containing myelin and other cellular debris was carefully removed using a Pasteur pipette and discarded. Fraction 2 containing the mitochondria was removed by pipetting with a 200 mL gel loading tip and diluted 1:4 in cold MIB and centrifuged at 14,000 rpm (15,000× *g*) for 10 min. The resulting pellet was then resuspended in 1 mL of MIB and centrifuged at 14,000 rpm (15,000× *g*) for 5 min. The final pellet was resuspended in 100 mL of MIB containing 10% 10 mg/mL bovine serum albumin.

### 4.10. Respiratory Chain Complexes Activity and ATP Content

The specific activity of NADH-decylubiquinone oxidoreductase (NQR) (complex I), succinate decylubiquinone DCPIP reductase (SQR) (complex II), and cytochrome c oxidase (complex IV) was measured using a Beckman Coulter spectrophotometer. Briefly, to disrupt the mitochondrial membranes for complex I measurement, 100 µg of mitochondrial suspension was first lysed by 3 min incubation in distilled water. Rotenone-sensitive Complex I activity was measured in assay medium with final volume of 1 mL (50 mM TRIS, pH 8.1, 2.5 mg/ mLBSA, 0.3 mM KCN, 0.1 mM NADH, 50 µM decylubiquinone without and with 3 µM rotenone) and followed the decrease in absorbance at 340 nm due to the NADH oxidation (ε = 6.22 mM^−1^ cm^−1^). Complex II activity was measured in 1 mL of assay medium containing 10 mM potassium phosphate pH 7.8, 2 mM EDTA, 1 mg/ mLBSA, 200 µg of mitochondrial protein, 0.3 mM KCN, 10 mM succinate, 3 µM rotenone, 0.2 mM ATP, 80 uM DCPIP (2,6-dichlorophenolindophenol), 1 µM Antimycin, and 50 µM decylubiquinone). The decrease in absorbance at 600 nm due to the oxidation of DCPIP at 600 nm (ε = 20.1 mM^−1^ cm^−1^) was recorded. Complex IV activity was measured by incubating 100 µg of mitochondrial protein in 1 mL of assay medium (40 mM potassium phosphate, pH 7.0; 1 mg/ mLBSA; 25 µM reduced cytochrome c) by following the oxidation of cytochrome c (II) at 550 nm (ε = 19.6 mM^−1^ cm^−1^). All assays were performed at 37 °C. FoF1-ATPase activity was measured following ATP hydrolysis with an ATP-regenerating system coupled to nicotinamide adenine dinucleotide phosphate (NADPH) oxidation, as previously reported [108]. Measurement of ATP concentration in the total homogenates from hippocampal area was performed by using a commercial bioluminescent assay kit (Sigma-Aldrich, St. Louis, MO, USA).

### 4.11. Seahorse XF Analyzer Respiratory Assay

Seahorse experiments were carried out in the HypACB facility at Sapienza Unviersity. Extracellular acidification rate (ECAR) and cellular oxygen consumption rate (OCR) were analyzed using XF Cell Mito Stress Test (Agilent) and measured by the extracellular flux analyzer XFe96 (Seahorse Bioscience, Houston, TX, USA). SHSY-5Y cells were cultured on XFe culture 96-wells miniplates (10 k/well) and treated following the same procedure illustrated in 4.2. First, 24 h before the experiment, the sensor cartridge for XFe analyzer was hydrated in a non-CO2 incubator at 37 °C. According to producer instructions, stressors concentrations were optimized and added as follows: 1 μM oligomycin as complex V inhibitor (2 μM for Col03), 1.5 μM FCCP (uncoupler agent), and 0.5 μM rotenone/antimycin A (complex I and III inhibitors). During sensor calibration, cells were incubated in non-CO_2_ incubator at 37 °C in 180 μLassay medium (XF base medium supplemented with 10 mM pyruvate, 10 mM glucose, and 2 mM L-glutamine (pH 7.4) was used to wash the cells and replace the growth medium); 30 min before Seahorse assay 100 nM of insulin (Humulin R, Ely-Lilly, Inadianapolis, IN, USA) was added if indicated. OCR was normalized for total protein/well/10k cells. Each sample/treatment was analyzed in 16 wells for experiment. Statistical analysis was executed on two independent experiments by exploiting one-way ANOVA followed by Bonferroni post hoc comparison test.

### 4.12. Statistical Analysis

Statistical analyses were performed using Student t test for the evaluation of differences between SD controls and the HFD group or CTR and IPA and using 1-way ANOVA for the comparison of CTR, Ins, IPA, and IPA Ins groups. Data are expressed as mean ± SEM or ± SD per group. All statistical analyses were performed using Graph Pad Prism 8.0 software (GraphPad, La Jolla, CA, USA).

## Supplementary Materials

The following are available online at https://www.mdpi.com/article/10.3390/ijms22073746/s1.
