# Supplementary material for: High-Fat Diet Leads to Reduced Protein O-GlcNAcylation and Mitochondrial Defects Promoting the Development of Alzheimer’s Disease Signatures
Source: Int J Mol Sci. 2021 Apr 3;22(7):3746. doi: 10.3390/ijms22073746 (PMC8038495; doi:10.3390/ijms22073746)
Supplement: Supplementary file 1 [file ijms-22-03746-s001.pdf]

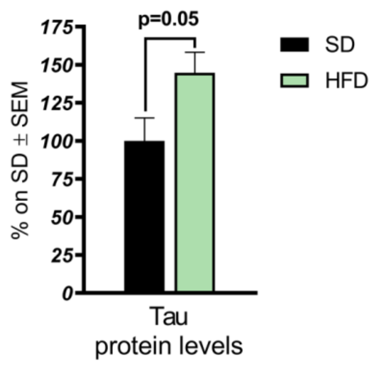

Figure S1: Quantification of tau protein expression leveles in the hippocampus of HFD mice in comparision to the SD-fed animals.

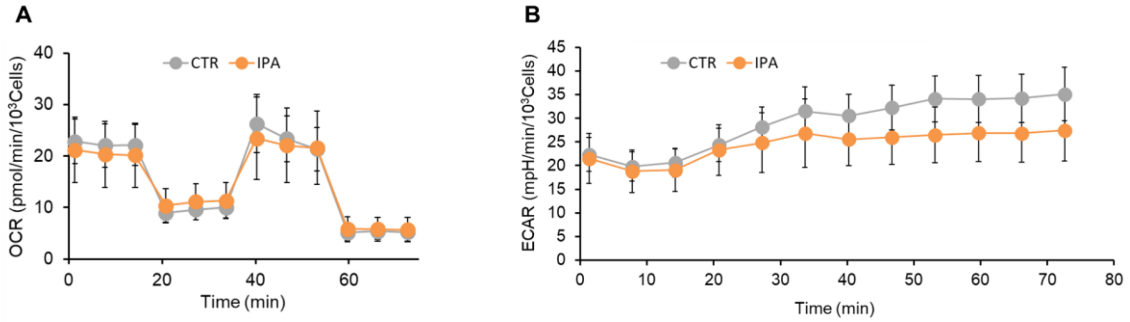

Figure S2: (A) Time-course of mitochondrial respiration obtained by OCR in IPA pre-treated cells and CTR. (B) Time-course of the glycolytic flux measured by ECAR in IPA pre-treated cells and CTR.

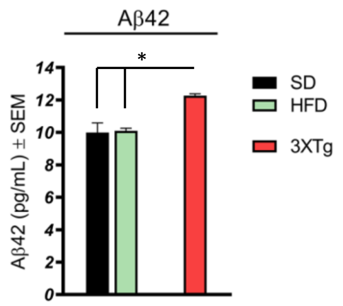

Figure S3: Evaluation of soluble Aβ 1-42 peptide by ELISA in the hippocampal region from SD and HFD animals. A comparable sample from 3XTg model was added as a positive control.
